# Pulmonary Sarcomatoid Carcinoma Initially Misdiagnosed as Aspergillosis: A Diagnostic Challenge

**DOI:** 10.1002/rcr2.70213

**Published:** 2025-05-19

**Authors:** YingYing Qian

**Affiliations:** ^1^ Department of Geriatrics, Second Affiliated Hospital, School of Medicine Zhejiang University Hangzhou China

**Keywords:** aspergillosis, chemotherapy, EGFR mutation, misdiagnosis, pulmonary sarcomatoid carcinoma

## Abstract

Pulmonary sarcomatoid carcinoma (PSC) is a rare and aggressive malignancy that often presents atypically, leading to incomplete diagnosis. We report a 55‐year‐old male with chronic obstructive pulmonary disease who presented with productive cough and haemoptysis. Initial bronchoscopy revealed fungal hyphae consistent with Aspergillus infection. The misleading clinical presentation led to an incomplete diagnosis. Subsequent lung biopsy and immunohistochemistry confirmed PSC harbouring an EGFR L858R mutation. Despite the presence of a targetable mutation, the patient showed a poor response to EGFR‐targeted therapy. This case highlights the diagnostic challenges and therapeutic complexities when PSC coexists with an airway fungal infection.

## Graphical abstract

A case of EGFR‐mutated pulmonary sarcomatoid carcinoma mimicking Aspergillus infection, leading to diagnostic delay. Despite harbouring a targetable mutation, the tumour showed poor response to EGFR‐TKI, highlighting the challenges of managing aggressive tumours with coexisting airway fungal infection.

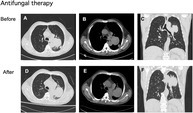

## 1 Introduction

PSC is a rare, aggressive subtype of non‐small cell lung cancer (NSCLC), accounting for less than 0.4% of all lung malignancies. It is characterised by a combination of epithelial and mesenchymal features, rapid progression, and poor response to conventional therapies [1]. Due to overlapping clinical and radiological features with infections, particularly fungal infections such as aspergillosis, PSC can be easily misdiagnosed [2]. Accurate and timely diagnosis relies on histopathological confirmation, which is often delayed when infectious etiologies are initially suspected. Here, we report a case initially diagnosed as pulmonary aspergillosis but subsequently confirmed as PSC, emphasising the diagnostic pitfalls and therapeutic dilemmas.

## 2 Case Report

A 55‐year‐old male smoker with a history of chronic obstructive pulmonary disease (COPD) presented with a 2‐month history of productive cough and haemoptysis. An initial chest computed tomography (CT) scan performed at a local hospital on 15 August 2018, revealed a cavitary lesion in the posterior segment of the left upper lobe, associated with bronchial obstruction and mildly enlarged mediastinal lymph nodes. Laboratory findings at that time were largely unremarkable except for a slightly elevated serum squamous cell carcinoma antigen (SCC, 4.2 ng/mL) and a positive serum (1 → 3)‐β‐d‐glucan (fungal G) test (189.30 pg/mL). Tuberculosis was excluded based on a negative T‐SPOT assay and clinical evaluation.

**FIGURE 1:**
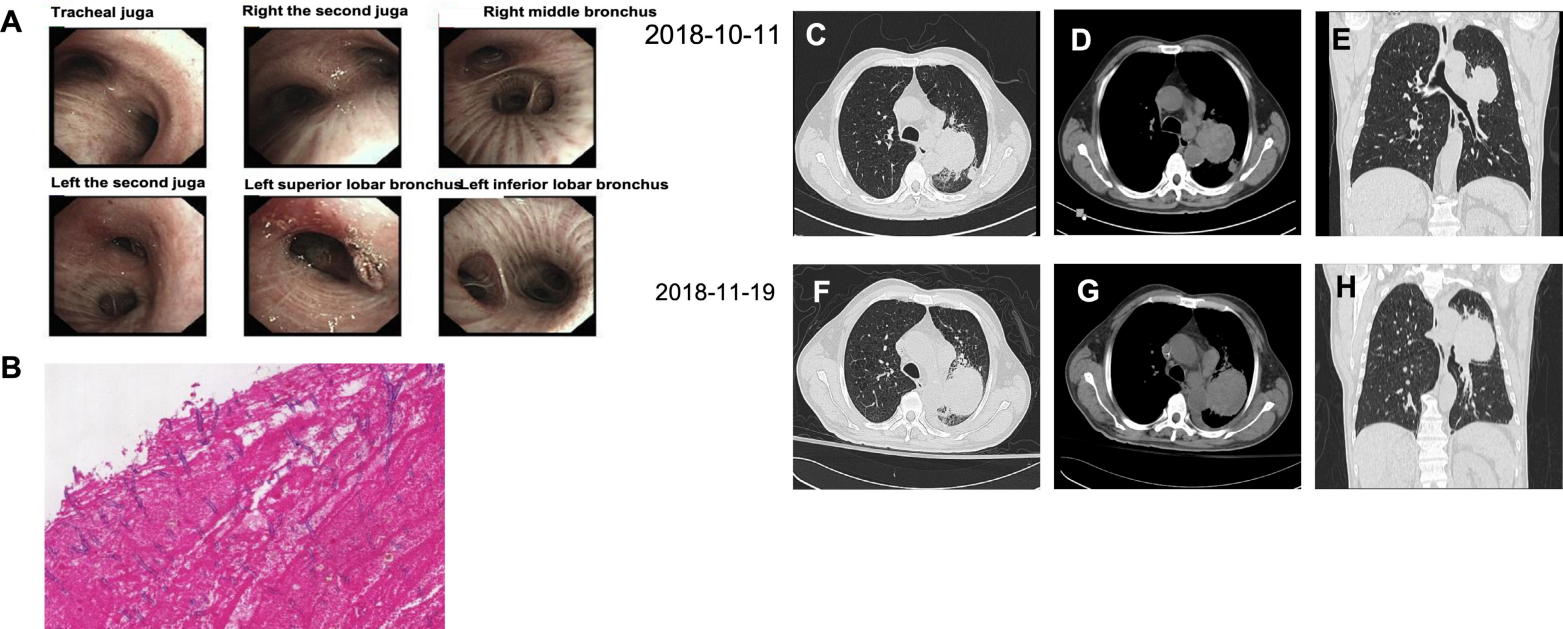
(A, B) Bronchoscopy and endobronchial biopsy. (C–H) Chest CT images showing tumour progression in the left lung following voriconazole therapy.

Bronchoscopic examination, performed shortly after the CT, revealed a mass completely obstructing the lumen of the left upper bronchus. Histopathological analysis of the initial biopsy specimen demonstrated inflammatory necrosis with the presence of Aspergillus hyphae. Taken together with the imaging findings of a cavitary nodule in the left upper lobe, these pathological results supported a preliminary diagnosis of pulmonary aspergillosis. The patient received oral voriconazole (200 mg twice daily) for 1 month, with partial symptomatic relief (Figure 1).

However, follow‐up CT showed significant tumour progression, measuring approximately 6.2 × 6.0 × 6.6 cm. The lesion had rapidly enlarged and evolved into a solid mass with heterogeneous internal density and mild heterogeneous enhancement on contrast‐enhanced imaging. These imaging features, combined with the rapid progression despite appropriate antifungal therapy, raised strong suspicion for malignancy. Subsequent PET/CT revealed hypermetabolic lesions in the lung (SUVmax 11.94) and right adrenal gland (SUVmax 15.51), indicating metastasis. CT‐guided biopsy confirmed PSC, staged as IV, with right adrenal metastasis.

**FIGURE 2:**
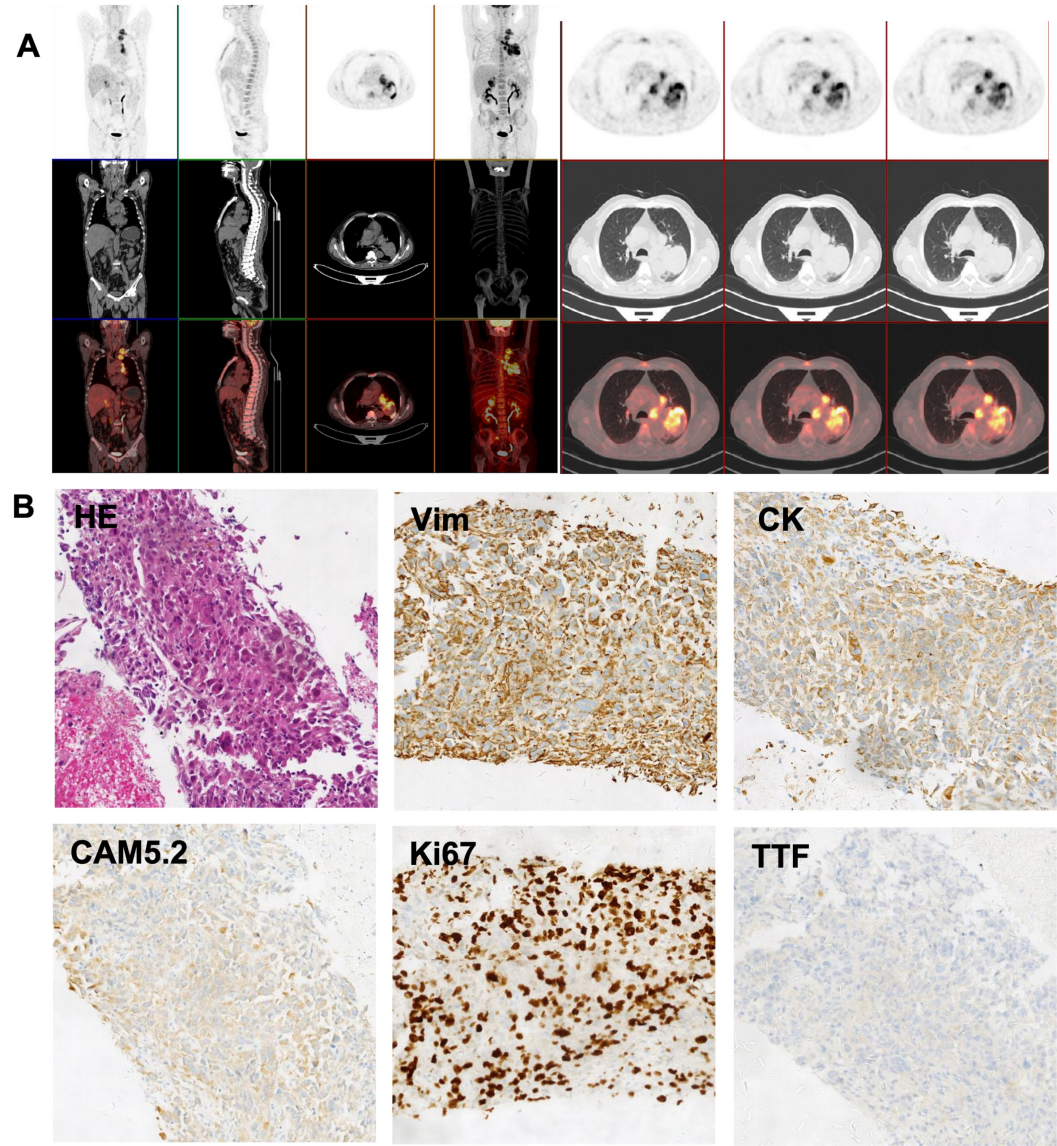
(A) Whole‐body PET/CT scan showing hypermetabolic lesions in the lung and adrenal gland. (B) Histopathological findings of the lung tumour. Sections stained with haematoxylin–eosin (HE) and immunohistochemically stained for vimentin, cytokeratin (CK), CAM5.2, Ki‐67, and TTF‐1. Magnification: ×100.

Immunohistochemistry was positive for CK (AE1/AE3), Cam5.2, and vimentin, and negative for CK5/6, p40, CD20, TTF‐1, and napsin A (Figure 2). Genetic testing identified EGFR (exon 21) and TP53 (exon 8) mutations (Table 1). Given the poor prognosis and treatment resistance of PSC, the patient initially received chemotherapy with paclitaxel and cisplatin but developed severe anaemia and fever after one cycle. Imaging showed progressive disease, prompting discontinuation of chemotherapy. Targeted therapy with icotinib (125 mg three times daily) was initiated based on EGFR mutation for approximately 20 days; however, the patient's condition rapidly deteriorated, developing refractory cachexia, persistent fever, and respiratory failure. With the informed consent of his family, aggressive treatments were discontinued, and the patient passed away shortly after discharge.

**TABLE 1:** Detection of mutations in the patient with pulmonary sarcomatoid carcinoma by next‐generation sequencing.

Gene	Exon	Reference sequence	Nucleotide change	Amino acid change
EGFR	exon21	NM_005228	c.2573T>G	p.L858R
TP53	exon8	NM_000546	c.844delC	p.R282fs
TMB (Tumour Mutation Burden)			7.88 Muts/MB	

## 3 Discussion

PSC is an uncommon and aggressive NSCLC variant, typically occurring in older male smokers. Radiologically, it often presents as a centrally located mass with cavitation or necrosis that closely resembles infections such as tuberculosis or fungal disease, often resulting in diagnostic delays [1]. These overlapping features contribute to frequent diagnostic delays or errors, as seen in our case.

There have been few reports of PSC complicated by invasive tracheobronchial aspergillosis. While pulmonary fungal infections are relatively common in lung cancer patients, airway‐localised fungal infections, particularly in PSC, are extremely rare. Unlike most reported cases where fungal infections occur after anticancer therapy, the airway infection in this patient developed prior to any treatment, likely due to tumour‐induced local immune dysfunction combined with COPD and frequent use of broad‐spectrum antibiotics and corticosteroids [3]. Additionally, although the patient harboured an EGFR L858R mutation, the presence of significant tumour heterogeneity and potential epithelial‐mesenchymal transition (EMT) likely contributed to the poor response to EGFR‐targeted therapy. The concurrent airway fungal infection further increased the complexity of treatment decisions.

Recent studies have reported EGFR mutations in a subset of PSC patients, with notable regional variation—up to 28% in Chinese populations, approximately 18% in Korean cohorts, and 3%–13% in Western countries [4]. Despite the presence of sensitive mutations such as L858R or exon 19 deletions, responses to tyrosine kinase inhibitors (TKIs) remain limited, likely due to intrinsic tumour heterogeneity. PSC typically exhibits biphasic histology, comprising both epithelial and sarcomatoid components, with the latter often demonstrating primary resistance to TKIs. Moreover, EMT, frequently observed in PSC, may further contribute to therapeutic resistance by promoting a mesenchymal phenotype with increased invasiveness and reduced drug sensitivity [5].

In addition to its structural heterogeneity, PSC is associated with a complex molecular landscape. High tumour mutational burden (TMB) and elevated PD‐L1 expression are commonly observed and may offer therapeutic opportunities with immune checkpoint inhibitors (ICIs) [5]. In our patient, the TMB was 7.88 mutations/Mb—slightly above the average for NSCLC but below the high TMB threshold (> 20 mutations/Mb) typically associated with favourable responses to ICIs. Although immunotherapy represents a promising strategy for PSC, further prospective studies are needed to define its clinical efficacy [6].

This case underscores the diagnostic and therapeutic challenges in managing PSC, particularly when complicated by coexisting fungal infection. To avoid misdiagnosis, clinicians should consider PSC in high‐risk individuals with non‐resolving cavitary lesions, even when infection is suspected. Early use of PET/CT and adequate biopsy sampling are critical. Multidisciplinary involvement—including pulmonology, pathology, radiology, and oncology—can facilitate timely diagnosis and guide individualised treatment strategies for this aggressive malignancy.

## Author Contributions

Y.Q. contributed to the study design, data collection, analysis, interpretation, and drafting and revision of the manuscript.

## Ethics Statement

This study was approved by the Research Ethics Committee of the Second Affiliated Hospital, School of Medicine, Zhejiang University (approval number: 2022‐1021).

## Consent

Written informed consent for publication of this case report and any accompanying images was obtained from the patient's daughter, as the patient is deceased.

## Conflicts of Interest

The author declares no conflicts of interest.

## Data Availability

The data that support the findings of this study are available on request from the corresponding author. The data are not publicly available due to privacy or ethical restrictions.
